# Case Report: IVCM of corneal chlorpromazine toxicity drug deposits

**DOI:** 10.3389/fmed.2025.1614699

**Published:** 2025-08-20

**Authors:** Xianwen Xiao, Xie Fang, Zhiwen Xie, Shunrong Luo, Yuan Lin, Huping Wu

**Affiliations:** ^1^Xiamen Eye Center and Eye Institute of Xiamen University, School of Medicine, Xiamen, China; ^2^Xiamen Clinical Research Center for Eye Diseases, Xiamen, Fujian, China; ^3^Xiamen Key Laboratory of Ophthalmology, Xiamen, Fujian, China; ^4^Fujian Key Laboratory of Corneal & Ocular Surface Diseases, Xiamen, Fujian, China; ^5^Xiamen Key Laboratory of Corneal & Ocular Surface Diseases, Xiamen, Fujian, China; ^6^Translational Medicine Institute of Xiamen Eye Center of Xiamen University, Xiamen, Fujian, China

**Keywords:** chlorpromazine, ocular surface, toxicity, corneal, drug deposits, *in vivo* confocal microscopy

## Abstract

To investigate corneal deposits in a patient undergoing long-term chlorpromazine therapy using *in vivo* confocal microscopy with the HRT II Rostock Corneal Module. We reported a 45-year-old woman with a 7-year history of chlorpromazine therapy presented with bilateral photophobia and a 4-year history of gradual-onset blurred vision. Slit-lamp examination revealed yellowish deposits in the corneal endothelium and Descemet’s membrane. *In vivo* confocal microscopy identified irregular hyper-reflective deposits in all corneal layers. The epithelial and superficial stromal deposits had well-defined edges, while the posterior stromal, Descemet’s membrane, and endothelial deposits appeared as hollow granules and streaks. This study is the first to use *in vivo* confocal microscopy to identify crystalline deposits in the corneal stroma and endothelium caused by high-dose chlorpromazine. These findings offer new insights into drug metabolism on the ocular surface and provide a basis for future research.

## Introduction

Chlorpromazine was the first antipsychotic drug to be successfully used to treat schizophrenia (1). Chlorpromazine primarily blocks dopamine D2 receptors in the brain, but it also binds muscarinic cholinergic, serotonin, and α1-adrenergic receptors, contributing to side effects (2). Excessive use may lead to sedation, cardiotoxicity, extrapyramidal side effects, and delirium.

Human corneal cells express a range of neuropeptides and neurotransmitters, including dopamine. In addition, these cells also express dopamine receptors, suggesting they are susceptible to stimulation by these substances within the cornea (3, 4). Dopamine is deaminated by monoamine oxidase and subsequently methylated by catechol-O-methyltransferase, undergoing metabolic degradation in the lacrimal glands and corneal epithelium (5). Chlorpromazine acts on dopamine receptors and can lead to stromal deposition in the cornea, typically appearing after the drug reaches a certain cumulative dose (6, 7).

Chlorpromazine deposits in ocular tissues when administered at high doses for prolonged periods. The most affected structures include the eyelids, conjunctiva, cornea, and crystalline lens (8, 9). The star-shaped deposits on the anterior lens capsule suggest that lens epithelial cells are migrating centripetally, accumulating the drug and eventually undergoing apoptosis. Additionally, convection currents in the aqueous humor may also contribute to this process. These deposits are well-documented to be dose-dependent and persist even after drug discontinuation. Here, we report ocular findings in a schizophrenia patient with a 7-year history of chlorpromazine use. It is the first report documenting such pronounced endothelial deposition at a particular cumulative dosage.

## Report of case

A 45-year-old woman with a 7-year history of schizophrenia was prescribed chlorpromazine (Contomin, Mitsubishi Pharma Corp., Osaka, Japan) at an initial dose of 125 mg/day. Over time, her dosage increased to as high as 1,000 mg/day, with a mean daily dose of 400 mg/day. According to the patient’s previous health examination records, uncorrected visual acuity (UCVA) prior to initiating chlorpromazine treatment was 0.6 in the right eye and 0.5 in the left eye, while best-corrected visual acuity (BCVA) was not measured. Approximately 3 years ago, the patient began to experience progressive visual decline, with bilateral UCVA gradually decreasing to 0.3. However, the patient refused further treatment at that time. Over the past 6 months, the blurring of vision has worsened significantly, prompting the patient to seek medical attention for further evaluation and management.

She presented with a gradual onset of blurred vision in both eyes. There was no significant ophthalmic or family history. Intraocular pressure (IOP) was measured at 11 mmHg in the right eye and 12 mmHg in the left eye. Upon examination, her uncorrected visual acuity was 0.15 in both eyes. In the right eye, best-corrected visual acuity reached 0.9 with a refraction of −2.50 diopters sphere (DS) / −1.50 diopters cylinder (DC). In the left eye, BCVA was 0.8 with a correction of −4.00 DS. The fundus examination was inconclusive due to media opacity.

**Figure 1:**
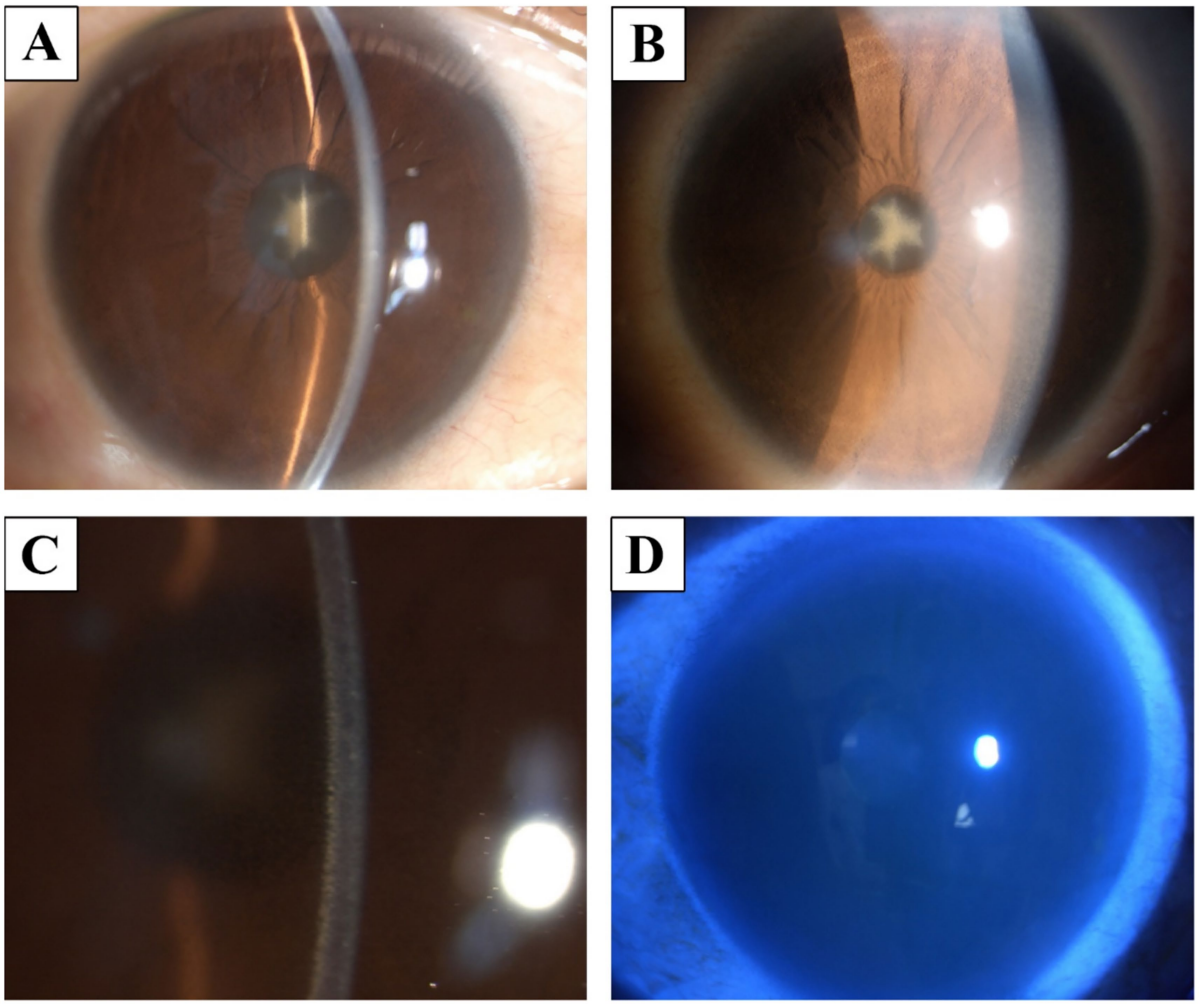
Slit-lamp finding. **(A,B)** a star-shaped opacity in the anterior lens capsule and nuclear sclerosis. **(C)** There was mild conjunctival hyperemia, with yellowish deposits on the corneal endothelium and Descemet’s membrane. **(D)** Corneal fluorescein staining showed no significant abnormalities.

Slit-lamp microscopy revealed a star-shaped opacity of the anterior lens capsule, and nuclear sclerosis was observed in both eyes (Figures 1A,B). Mild bilateral conjunctival hyperemia was noted, along with yellowish deposits primarily on the corneal endothelium and Descemet’s membrane (Figure 1C). The anterior chambers appeared calm. Fluorescein sodium staining revealed no apparent corneal epithelial defects (Figure 1D).

**Figure 2:**
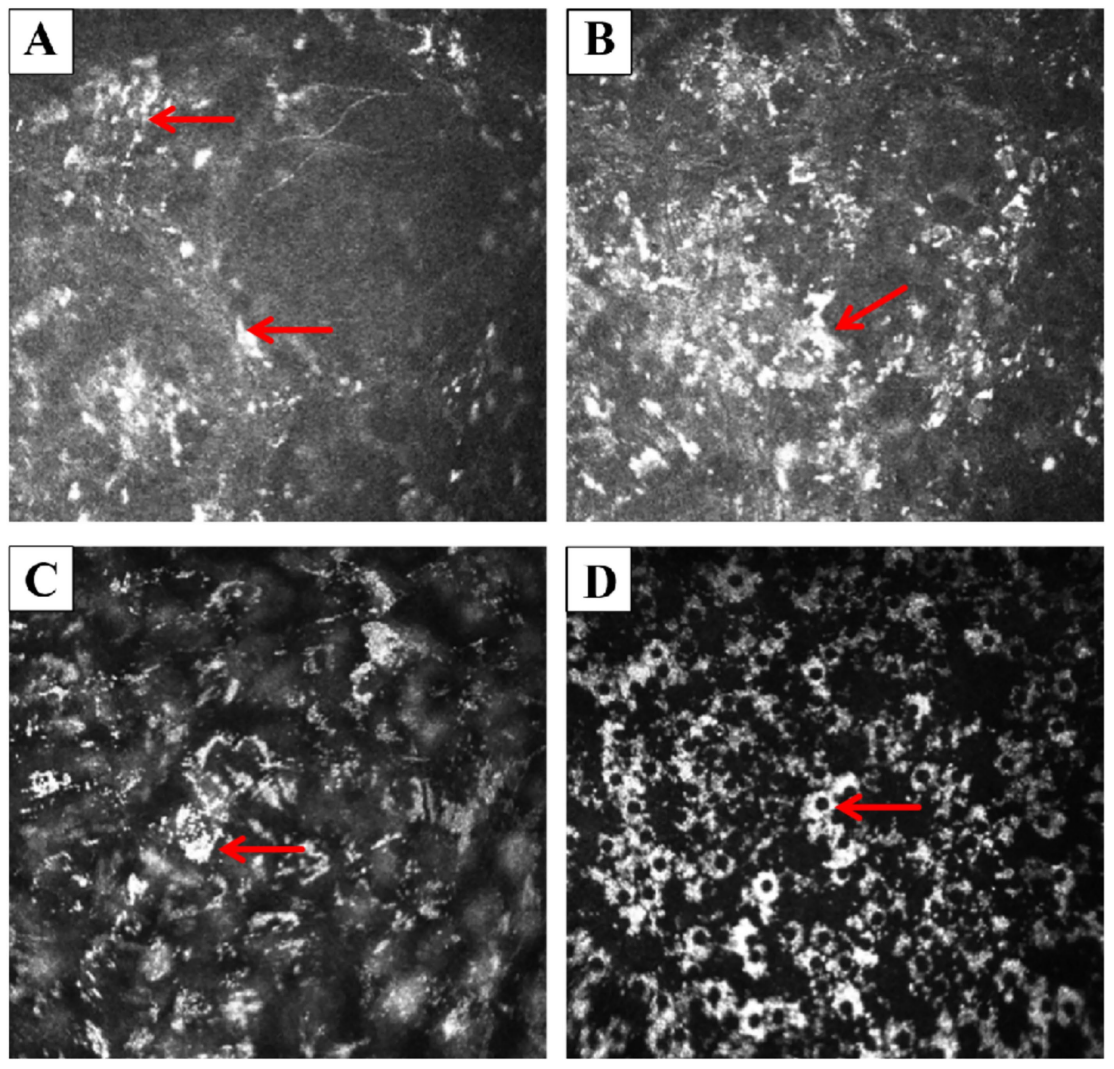
In vivo corneal images obtained by the HRT II RCM. **(A)** Epithelium: multiple small, discrete hyperreflective deposits were scattered across the epithelial layer. A well-demarcated, block-like white deposit was observed (arrow). **(B)** Anterior stroma: the deposits appeared to coalesce into larger, confluent plaques (arrow), with surrounding punctate hyperreflective dots scattered irregularly in the stromal background. **(C)** Posterior stroma (near Descemet’s membrane): the stroma exhibited a diffuse, unevenly reflective “smoky” appearance throughout the full thickness. Crystalline-like scattered deposits adherent to the posterior stroma were noted (arrow). **(D)** Endothelium: endothelial cells were faintly visible and exhibited relatively preserved, low-reflective morphology. Numerous suspended, ring-shaped or “silver-halo”-like hyperreflective deposits consistent with keratic precipitate-like drug accumulations were present (arrow).

**Figure 3:**
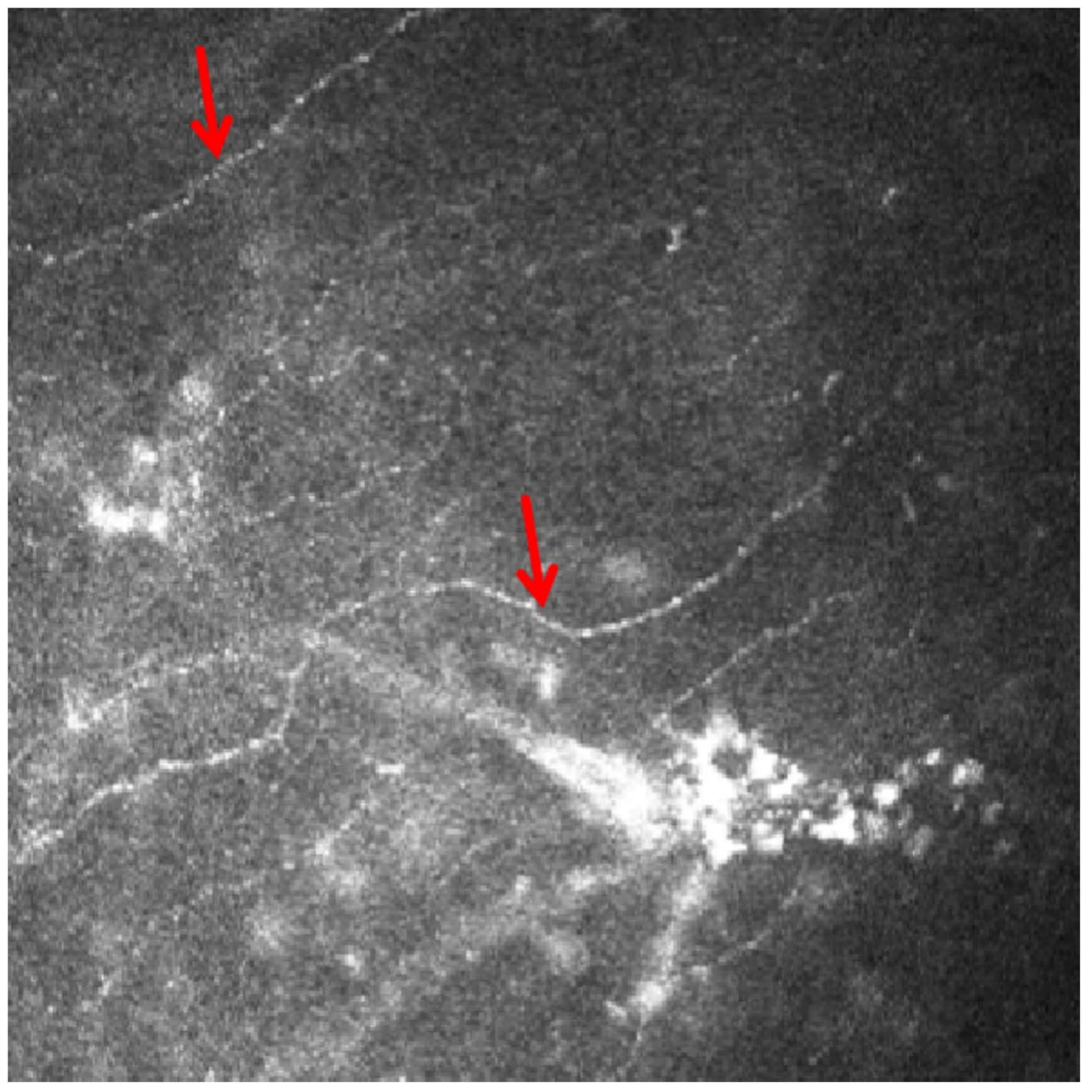
The IVCM images of the subbasal nerve plexus revealed fragmented and thinned nerve fibers (arrow), with a markedly reduced density and sparse distribution throughout the examined area.

Corneal endothelial cell count could not be measured by the HRT II Rostock corneal module (RCM), as deposits heavily affected the endothelium (Figure 2). RCM revealed multilayered corneal abnormalities consistent with chlorpromazine-induced toxicity. At the subbasal nerve plexus layer, there were scattered hyperreflective deposits accompanied by a marked reduction in nerve fiber density, and the remaining nerves appeared short, fragmented, and barely visible amidst the deposits (Figure 3). In the anterior stroma, numerous punctate and granular hyperreflective deposits were irregularly distributed, indicating chlorpromazine accumulation at this level. The mid-stromal layer showed dense, coarse, and heterogeneous hyperreflective aggregates, along with localized stromal disorganization. At the endothelial level, characteristic “target-like” or “ring-shaped” hyperreflective deposits were observed, suggestive of drug-induced endothelial toxicity.

The patient was referred for psychiatric evaluation with the recommendation to reduce the dosage of the implicated medication. However, additional investigations were not completed due to socioeconomic limitations and suboptimal follow-up adherence. Through telephone and electronic follow-up, it was noted that 1 year after the adjustment of the medication dosage, the patient’s UCVA had improved to 0.4 in the right eye and 0.3 in the left eye. The patient was subsequently lost to follow-up.

## Discussion

Long-term use of chlorpromazine can result in dose-dependent deposits in the eyes that remain even after the medication is stopped. In this patient, deposits were found in the corneal epithelium and the superficial stroma, with a predominance in the posterior stroma, Descemet’s membrane, and endothelium. Dopamine D2 receptors have been identified in both the corneal endothelium and epithelium, which may account for the significant deposition observed in the endothelial layer of this patient (10). This indicates that chlorpromazine likely enters the aqueous humor before being deposited in the eye, and exposure to sunlight may exacerbate its ocular toxicity (11).

Chlorpromazine is known to cause blue-gray skin pigmentation, especially in areas exposed to sunlight, affecting about 1 to 2.9% of patients on long-term psychiatric treatment (12). Lenticular changes are seen in 50% of patients who have received a cumulative dose greater than 1,000 g, while corneal and conjunctival changes are more likely to occur at higher doses (13). Possible mechanisms behind these effects include the formation of chlorpromazine photoadducts with DNA, which can lead to DNA damage from photosensitization, or the oxidation of its metabolite, 7-hydroxychlorpromazine, which produces a substance similar to melanin (14). In our patient, there were no signs of conjunctival changes or abnormal skin pigmentation, likely due to the cumulative dose she received.

This case study has a key limitation. The current evidence is insufficient to establish a direct causal relationship between chlorpromazine intake and the onset of blurred vision. We were unable to assess the expression of dopamine D2 receptors in the patient’s corneal tissue through *in vivo* analysis or histological examination. Future studies are needed to further investigate this potential mechanism using molecular biology techniques or advanced imaging methods.

*In vivo* confocal microscopy previously showed endothelial pleomorphism and polymegethism, likely due to chlorpromazine phototoxicity (15). However, in this case, endothelial deposits were so dense that cell morphology was unobservable. The HRT II RCM imaging system successfully identified corneal deposits that were indistinct on slit-lamp microscopy, highlighting its potential for detecting early drug-induced ocular toxicity.

## Data Availability

The original contributions presented in the study are included in the article/supplementary material, further inquiries can be directed to the corresponding authors.
